# A preclinical orthotopic model for glioblastoma recapitulates key features of human tumors and demonstrates sensitivity to a combination of MEK and PI3K pathway inhibitors

**DOI:** 10.1242/dmm.018168

**Published:** 2014-11-27

**Authors:** Rajaa El Meskini, Anthony J. Iacovelli, Alan Kulaga, Michelle Gumprecht, Philip L. Martin, Maureen Baran, Deborah B. Householder, Terry Van Dyke, Zoë Weaver Ohler

**Affiliations:** 1 Center for Advanced Preclinical Research, Leidos Biomedical Research, Inc, Frederick National Laboratory for Cancer Research, Frederick, MD 21702, USA.; 2 Mouse Cancer Genetics Program, Frederick National Laboratory for Cancer Research, Frederick, MD 21702, USA.

**Keywords:** Glioblastoma, Mouse model, PI3K and MEK inhibition, Apoptosis

## Abstract

Current therapies for glioblastoma multiforme (GBM), the highest grade malignant brain tumor, are mostly ineffective, and better preclinical model systems are needed to increase the successful translation of drug discovery efforts into the clinic. Previous work describes a genetically engineered mouse (GEM) model that contains perturbations in the most frequently dysregulated networks in GBM (driven by RB, KRAS and/or PI3K signaling and PTEN) that induce development of Grade IV astrocytoma with properties of the human disease. Here, we developed and characterized an orthotopic mouse model derived from the GEM that retains the features of the GEM model in an immunocompetent background; however, this model is also tractable and efficient for preclinical evaluation of candidate therapeutic regimens. Orthotopic brain tumors are highly proliferative, invasive and vascular, and express histology markers characteristic of human GBM. Primary tumor cells were examined for sensitivity to chemotherapeutics and targeted drugs. PI3K and MAPK pathway inhibitors, when used as single agents, inhibited cell proliferation but did not result in significant apoptosis. However, in combination, these inhibitors resulted in a substantial increase in cell death. Moreover, these findings translated into the *in vivo* orthotopic model: PI3K or MAPK inhibitor treatment regimens resulted in incomplete pathway suppression and feedback loops, whereas dual treatment delayed tumor growth through increased apoptosis and decreased tumor cell proliferation. Analysis of downstream pathway components revealed a cooperative effect on target downregulation. These concordant results, together with the morphologic similarities to the human GBM disease characteristics of the model, validate it as a new platform for the evaluation of GBM treatment.

## INTRODUCTION

High-grade astrocytomas, including glioblastoma multiforme (GBM), are the most common malignant brain tumors, and current therapies are largely ineffective (Belda-Iniesta et al., 2008; DeAngelis, 2001). Surgical tumor resection followed by radiation therapy and/or temozolomide treatment is the standard of care for GBM (Becker and Yu, 2012; Stupp et al., 2005); however, results are modest, and the median survival is approximately 14 months post-diagnosis (Becker and Yu, 2012; Maher et al., 2001; Stewart, 2002; Stupp et al., 2005). Recently, more detailed analyses of the molecular pathogenesis of GBM have pointed to the potential for using molecularly targeted agents in the treatment of the disease (Tanaka et al., 2013).

A report from The Cancer Genome Atlas project (TCGA) identified the most commonly dysregulated pathways in GBM (TCGA, 2008). The receptor tyrosine kinase (RTK)-RAS-phosphoinositide 3-kinase (PI3K) pathway is activated in 88% of GBM tumors (Guha et al., 1997; TCGA, 2008), and in lower grade gliomas, activation is predictive of a poor outcome (McBride et al., 2010). In 36% of cases, PI3K signaling is further dysregulated by phosphatase and tensin homolog (PTEN) deficiency (TCGA, 2008), whereas inactivation of the p53 and RB tumor suppressor pathways is characteristic of 87% and 78% of GBM tumors, respectively. Concurrent gene aberrations in the three pathways are implicated in 74% of GBM pathogenesis (TCGA, 2008), indicating that targeting a single receptor might be less than optimal as a therapeutic regimen.

Recently, a genetically engineered mouse (GEM) model has been developed that harbors perturbations in RTK, PI3K, and RB networks, and develops spontaneous p53 aberrations, indicating potential genomic diversity within tumors (Miller et al., 2009; Schmid et al., 2012; Song et al., 2013; Vitucci et al., 2013). The GEM-GBMs initiate in adult glial fibrillary acidic protein (GFAP)-expressing glial progenitor populations (mainly astrocytes), with dominant inactivation of RB tumor suppression, and progress to GBM upon induction of the constitutively active mutant KRAS^G12D^ and deletion of PTEN alleles, followed by spontaneous somatic p53 missense mutations. These engineered transforming mutations result in major alterations of three signaling pathways that are crucial to human GBM (TCGA, 2008). Therefore, this model represents various human GBM subtypes, in which common downstream targets of RTKs are activated. Based on the engineered alleles, induced animals are referred to as ‘TRP’ mice. TRP mice develop diffuse, low-grade astrocytomas following induction, which is followed by progression to high grade astrocytomas that are histologically similar to human GBM after 4 to 6 months (Miller et al., 2009; Motomura et al., 2012; Schmid et al., 2012). Comparable to human GBM, these GEM tumors display linear foci of necrosis with pseudopalisading by neoplastic cells and dense vascularization. However, the long latency to tumorigenesis and heterogeneity in the timing of advanced tumor development makes the use of the TRP GEM model in preclinical studies challenging.

**TRANSLATIONAL IMPACT**

**Clinical issue**

Glioblastomas (GBM) are the most common malignant brain tumors, and despite progress in elucidating the molecular and pathological basis of tumor development, current treatment strategies are largely ineffective. The predominant adjuvant therapy following surgical tumor resection relies on DNA damage induced by radiation and/or temozolomide treatment. Animal models of GBM are essential for the evaluation of more efficacious molecularly targeted therapies, in particular whether they can faithfully replicate both the relevant signaling pathway alterations and the histopathological hallmarks of human GBM.

**Results**

In this study, brain tumor cells from genetically engineered mice carrying activating mutations in the retinoblastoma protein (RB), receptor tyrosine kinase (RTK-RAS) and phosphatase and tensin homolog (PTEN) networks (the major signaling pathways altered in human GBM) were injected orthotopically into the brains of immune-competent, genetically identical background strains of mice in order to generate a GBM model. This tractable and reliable model retains key characteristics of the human disease, including vascularity and aggressive invasion into surrounding tissue, and an intact immune system. Sensitivity to targeted drugs was examined by testing the inhibition of phosphoinositide 3-kinase (PI3K) and mitogen-activated protein kinase (MAPK) pathways, both *in vitro* in primary cultures and *in vivo* in this model, by employing drugs that are currently in clinical trials for GBM. Neither the PI3K inhibitor BKM120 nor the MEK inhibitor PD0325901 given as single agents significantly improved mouse survival. However, combination therapy led to an increase in cancer cell apoptosis, a decrease in tumor cell proliferation and increased survival owing to a synergistic effect of the two drugs on suppression of the PI3K pathway (which regulates cell proliferation and survival).

**Implications and future directions**

The mouse model described here allows for the examination of targeted therapies on pathways that are perturbed in GBM. Human GBMs harbor amplification in multiple receptor tyrosine kinase genes; therefore, this model is representative of various tumor subtypes in which common downstream genes are activated. The combination of PI3K and MEK inhibitors has the potential to control GBM tumor growth and extend survival, yet more studies are needed to optimize beneficial effects and reduce resistance and/or toxicity in these targeted treatments. Remarkably, the orthotopic tumors are highly proliferative, invasive and vascular. The aggressive nature of GBM in this model, as well as its molecular and histopathological features, warrant continued use for improving upon existing therapeutic strategies, as well as for testing novel targeted drug treatments or immunotherapy approaches.

Given the devastating nature of GBM in the clinic and the lack of effective therapies, a preclinical model is needed that recapitulates both the histopathological and molecular features of human GBM, and that is conducive to drug-efficacy analysis within a reasonable time frame (Hambardzumyan et al., 2011; Huse et al., 2013). Standard xenograft models lack the key histologic features that are characteristic of human GBM, as well as the immune system component (Becher and Holland, 2006). Moreover, a good model should represent the highly proliferative and infiltrative nature of GBM, including microvascular proliferation and areas of focal necrosis (Wen and Kesari, 2008).

Here, we describe a more tractable model for the preclinical evaluation of therapies for GBM. We developed a GEM-derived orthotopic model using primary brain tumor cells that had been isolated from induced TRP mice. Primary cells injected orthotopically into immune competent syngeneic mouse brains induced grade IV tumors within 2 to 3 weeks and recapitulated TRP GEM tumor histopathology, including its invasive properties. We examined the effect of targeted kinase inhibitor drugs on the PI3K and mitogen-activated protein kinase (MAPK) pathways using primary tumor cells *in vitro*, and we found that drug combinations targeting both pathways resulted in a substantial cytotoxic effect. Although *in vitro* cell proliferation was sensitive to PI3K inhibitors, feedback loops and incomplete pathway suppression require the addition of MEK inhibitors to achieve significant tumor cell death *in vivo*. We demonstrate that this model is valuable for examining targeted-drug responses in GBMs and for elucidating the survival benefit of different treatment regimens. Our results show that combined targeting of the PI3K and MAPK pathways can have a synergistic effect in glioblastoma, highlighting the relevance of our preclinical model for further studies in order to refine therapeutic regimens.

## RESULTS

### Key histopathological features of the GBM engineered mouse model translate to the orthotopic model

Before developing a TRP-GEM-derived orthotopic model, we evaluated the histopathology of grade IV GBM tumors from the *de novo* GEM (supplementary material Fig. S1A). High-grade astrocytoma developed 4 to 5 months post-induction with tamoxifen and recapitulated key features of human GBM (Hadjipanayis and Van Meir, 2009; Miller et al., 2009; Schmid et al., 2012). Tumors were highly cellular, invasive and pleomorphic with a high mitotic rate, exhibiting linear to serpentine foci of necrosis with pseudopalisading by neoplastic cells. They also displayed increased vascularization with numerous small tortuous irregular dilated blood vessels, thrombosis and hemorrhage (supplementary material Fig. S1B, H&E). For comparison with cellular markers that are characteristic of human GBM (Motomura et al., 2012; TCGA, 2008), we examined the level and distribution of GFAP, Nestin, Sox-2 and Olig-2 in GBM from TRP mice by immunohistochemistry (IHC) (supplementary material Fig. S1B,C). The distribution of the neural progenitor markers Nestin and Sox-2 (supplementary material Fig. S1C) aligned with other models in that Nestin expression was cytoplasmic and mainly in the tumor periphery, whereas Sox-2 was expressed in the nucleus by the majority of proliferative cells (Alcantara Llaguno et al., 2009; Kwon et al., 2008; Prestegarden et al., 2010). Olig-2, which promotes proliferation in neural progenitor cells and is a marker for differentiated glial cells in normal central nervous system tissue (Alcantara Llaguno et al., 2009; Kwon et al., 2008), was highly expressed in TRP tumor tissue (supplementary material Fig. S1C). Most proliferative cell clusters express Olig-2 in grade IV tumors. T121 was expressed in the majority of tumor cells, indicating widespread inactivation of RB (supplementary material Fig. S1B). Overall, GBM tumors from TRP mice expressed neuronal and glial progenitor markers, as described for human GBM (Motomura et al., 2012), suggesting that the cellular heterogeneity observed in human disease is conserved, and a more tractable model derived from the GEM would be of value for preclinical assays.

**Fig. 1.**
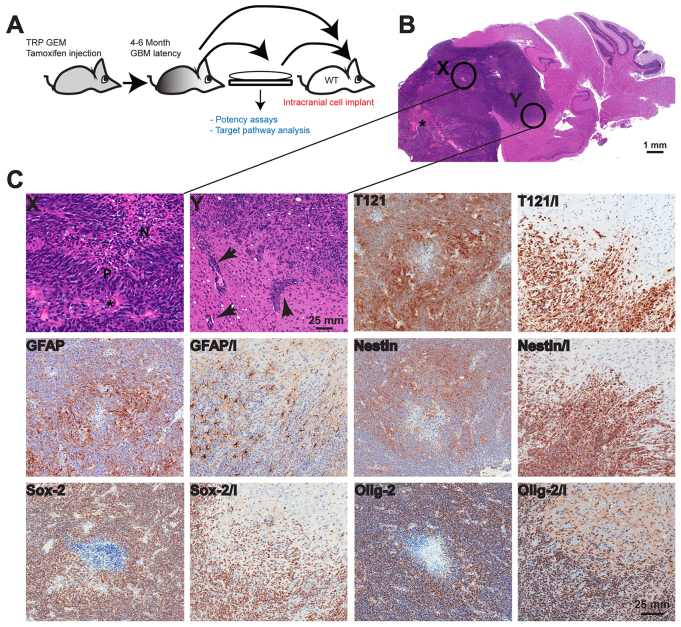
**Orthotopic GBM model characterization.** (A) Tumor cells were isolated from TRP grade IV astrocytoma and cultured for several passages prior to intracranial injection into syngeneic mouse brains, or cells were implanted directly. (B) Both methods resulted in grade IV astrocyomas. Orthotopic tumors have large numbers of variably sized irregular blood vessels (*). Enlarged images of areas labeled X and Y are shown in panel C. (C) Orthotopic GBMs feature necrotic foci (N in region labeled X) in central regions that are lined by pseudopalisading tumor cells (P). The invasive tumor cells often track along adjacent small blood vessels (arrows in region labeled Y), as well as diffusely invading through the recipient’s neuropil. Two images are shown for each marker stain; in the second, invasion is indicated by ‘I’ where orthotopic tumors contain foci of extensive invasion into the adjacent recipient normal brain. Tumor cells express variable levels of T121 with increased levels at the invasive edge highlighting individual invading tumor cells (T121 and T121/I). GFAP expression is heterogeneous with greater numbers of negative cells at the periphery (GFAP and GFAP/I). Nestin expression is also greater at the invasive front (Nestin and Nestin/I). Most tumor cells express Olig-2 (Olig-2 and Olig-2/I) and Sox-2 (Sox-2 and Sox-2/I).

To establish an orthotopic mouse model for GBM, tumor cells were isolated from TRP grade IV astrocytoma and cultured for at least two passages before intracranial injection into syngeneic mouse brains. Cells were also injected directly without culture, and both methods resulted in grade IV astrocytomas that recapitulated the tumor histopathology of *de novo* TRP GBM (Fig. 1A). The highly proliferative, invasive and vascular features of the orthotopic grade IV tumors correlated with histopathology features that define human GBM, including necrosis and pseudopalisading by neoplastic cells (Fig. 1B,C). The staining pattern for Nestin was similar to that observed in the GEM, with expression in invasive cells and intense staining around the necrotic tumor area (Fig. 1C, Nestin and Nestin/I). Sox-2, Olig-2 and GFAP were present at high levels in proliferative cells and expressed in the invasive part of the tumor, and T121 staining confirmed suppression of RB (Fig. 1, Sox-2, Sox-2/I, Olig-2, Olig-2/I, T121, T121/I and GFAP, GFAP/I). Hence, the GBM orthotopic model retained the key features of *de novo* TRP glioblastoma tumors while recapitulating human GBM histopathology in an immunocompetent background (Motomura et al., 2012; TCGA, 2008).

### GBM-derived cells are sensitive to drugs that target the PI3K or MAPK pathways

In addition to establishing the orthotopic tumor model, we derived primary tumor cells from TRP GBM as a tool for drug *in vitro* screening before *in vivo* evaluation. We first examined the standard chemotherapy agents temozolomide, doxorubicin and paclitaxel in cell viability assays. The GBM-derived cells were resistant to temozolomide [half maximal effective concentration (EC_50_)>50 μM], the clinical standard of care along with radiation (Becker and Yu, 2012; Stupp et al., 2005), but sensitive to doxorubicin (EC_50_=9 nM) and paclitaxel (EC_50_=2 nM) (supplementary material Fig. S2). However, both doxorubicin and paclitaxel have limited efficacy in individuals as a result of their inability to penetrate the blood-brain barrier (Baumann et al., 2013; Verreault et al., 2011).

**Fig. 2.**
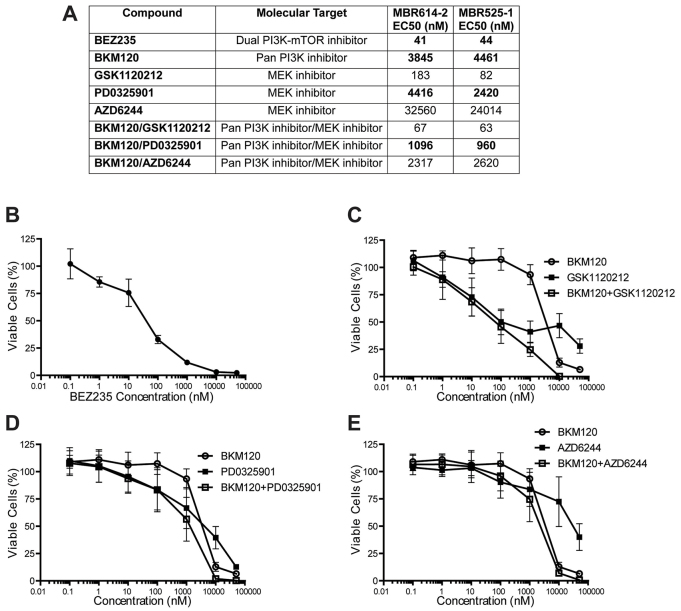
**Potency of targeted drugs in GBM-derived primary cells**. Primary GBM cells were treated with increasing concentrations of BEZ235, BKM120, GSK1120212 or PD0325901 alone or in combination at equimolar drug concentrations. XTT assays were performed as described in Materials and Methods. (A) Summary table shows the effects of different drug treatments on GBM cell viability as EC_50_ (nM); EC_50_ of compounds used for *in vivo* efficacy studies are in bold. (B-E) Representative MBR614-2 GBM cell viability plots, after 72 h of treatment, as a percentage of the vehicle-treated control cells. Each single and combination drug test was repeated at least three times in three independent experiments. Each dose treatment was performed in triplicate and bars represent s.d.

As MAPK and PI3K signaling is aberrant in TRP GBM tumors, we evaluated two primary cell lines for their sensitivity to inhibitors targeting intermediates in those pathways. Both lines were more sensitive to the dual PI3K-mTOR inhibitor BEZ235 (EC_50_ of 41 nM and 44 nM) than to the PI3K inhibitor BKM120 alone (EC_50_ of 3845 nM and 4461 nM) (Fig. 2A–C). The cells displayed varying sensitivity to MEK inhibitors, with EC_50_ values of 183 nM and 82 nM for GSK1120212, 4.4 μM and 2.4 μM for PD0325901, and 32 μM and 24 μM for AZD6244 (Fig. 2A,C–E). Cell growth was not completely inhibited by AZD6244, even at concentrations up to 50 μM (Fig. 2A,E). We therefore examined the growth inhibitory effect of combining the PI3K and MEK inhibitors at equimolar concentrations. Both primary cell lines were more sensitive to BKM120 in combination with GSK1120212, PD0325901, or AZD6244 than any of the compounds alone, and they both exhibited complete growth inhibition at higher concentrations (Fig. 2A,C–E), indicating the potential for additive or synergistic compound effects.

### MEK inhibition enhances suppression of the PI3K pathway *in vitro*

**Fig. 3.**
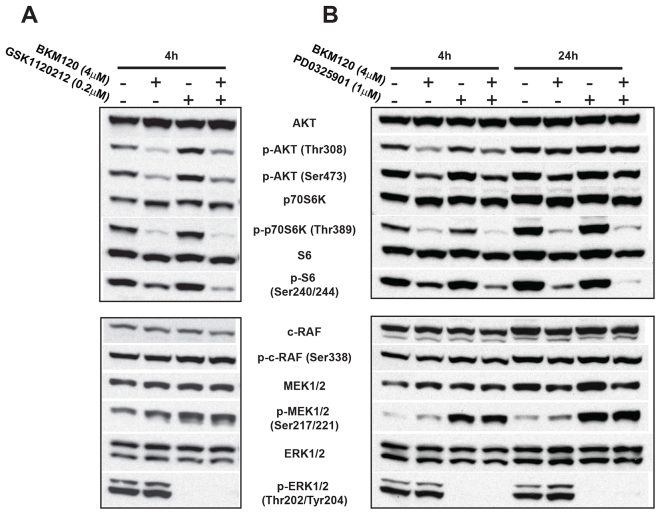
**Combined treatment of GBM cells results in enhanced response *in vitro*.** Drug target response in the PI3K and MAPK pathways was evaluated by immunoblotting after 4 h and 24 h of treatment. Drug concentrations were based on the EC_50_ determined in cell viability assays (Fig. 2). (A) MBR614-2 cells were treated with BKM120 (4 μM) and/or GSK1120212 (0.2 μM). Only the combined treatment resulted in a reduction of p-S6. (B) Cells were treated with BKM120 (4 μM) and/or PD0325901 (1 μM). Reduction of p-S6 was greatest with the drug combination after 24 h.

To confirm that compound potency corresponded to target inhibition, we examined the phosphorylation of target proteins in GBM primary cells MBR614-2 after 4 or 24 h of drug exposure (supplementary material Fig. S3A; Fig. 3). In cells that had been treated with BEZ235, phosphorylation of AKT at both residues Thr308 and Ser473 was decreased (p-AKT/Thr308 and p-AKT/Ser473, respectively), resulting in a reduction of phosphorylated S6 (p-S6), as previously described for human glioma and lung cancer cells (Herrera et al., 2011; Liu et al., 2009). Interestingly, expression of phosphorylated MEK (p-MEK) was increased after treatment without a corresponding increase in phosphorylated ERK (p-ERK) after 4 h of treatment (supplementary material Fig. S3A).

BEZ235 was more potent in cell viability assays than the PI3K-MEK inhibitor combination (Fig. 2A), yet BEZ235 did not fully reduce p-AKT; therefore, we hypothesized that the combination treatment might be more effective in inducing sustained suppression of both the PI3K and MAPK pathways. We found that BKM120 alone reduced the expression of p-AKT/Thr308/Ser473 and phosphorylated p70S6K (p-p70S6K), but had only a slight effect on p-S6 (Fig. 3A,B). A slight induction of p-MEK1/2, but not p-ERK, with BKM120 paralleled the response to BEZ235 (Fig. 3A,B; supplementary material Fig. S3A). The MEK inhibitors GSK1120212 and PD0325901 both effectively reduced p-ERK in GBM cells, with a corresponding induction of p-MEK expression (Fig. 3A,B). This feedback induction of p-MEK was not accompanied by an upstream increase in phosphorylated c-RAF (p-c-RAF) levels, nor did it affect the suppression of p-ERK. BKM120 in combination with either GSK1120212 or PD0325901 resulted in the suppression of p-AKT, p-p70S6K, and p-S6, as well as p-ERK; moreover, the reduction of S6 phosphoprotein was enhanced relative to treatment with BKM120 alone and was even more pronounced after 24 h. This enhanced target response is consistent with the improved growth inhibition that we observed using the drug combinations in cell-based assays (Fig. 2C,D).

### Time and concentration-dependent profiling of PI3K and MAPK inhibitors

**Fig. 4.**
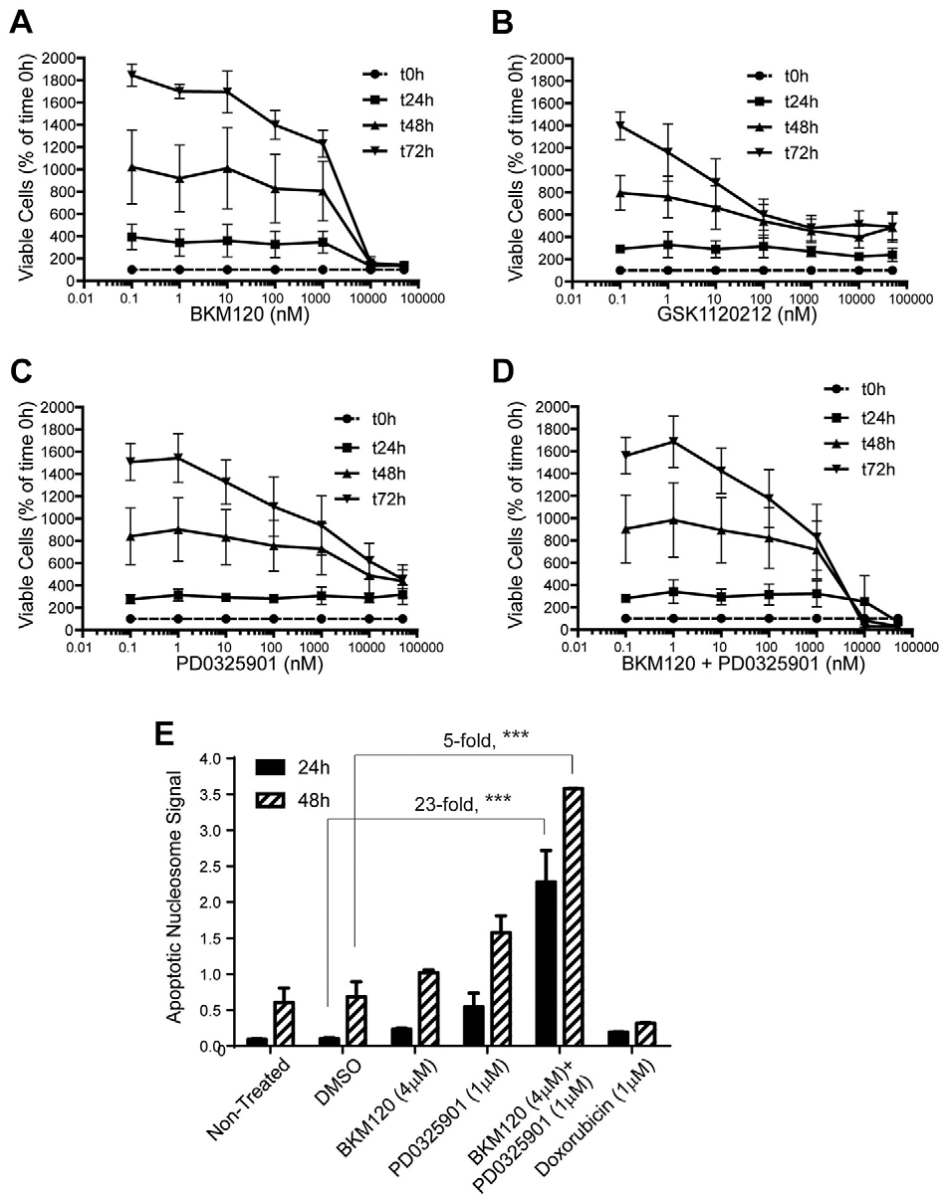
**Profiling GBM cell sensitivity to PI3K and MEK inhibitors.** (A-D) Cells were exposed to the indicated drug concentrations for 24, 48 or 72 h and cell growth and viability (by XTT assays) were determined by normalization to the viability at time 0 h (dashed curve line). (E) Apoptosis assay in GBM cells. Cells were treated with DMSO, BKM120 (4 μM), PD0325901 (1 μM) or a combination (BKM120+PD0325901) for 24 or 48 h. The combination resulted in a greater increase in cell death up to 23-fold at 24 h and fivefold at 48 h over the level of that of DMSO controls. Each treatment was performed in triplicate (bars represent the s.d., ***P<0.0001). Doxorubicin treatment was included in the assay as a cytotoxic control.

To further evaluate the relative effect of inhibitors either alone or in combination on the net growth of GBM cells, we conducted timed cell proliferation assays with MBR614-2 cells (Fig. 4A–D). After drug exposure for 24, 48 or 72 h, XTT viability assays were used to determine cell viability relative to that of DMSO-treated cells at time 0. At the EC_50_ for BKM120 (4000 nM), MBR614-2 percent cell viability was reduced to the level of time 0 as early as 24 h, indicating growth inhibition (Fig. 4A). Still, the maximum response to BKM120 was cytostatic, as cell proliferation was inhibited without net cell loss for up to 72 h. By contrast, cell viability was 50% of the viability at time 0 after 48 h of exposure to BEZ235 (supplementary material Fig. S3B). Viability was not decreased to the levels at time 0 at the 24- to 72-h time points with either GSK1120212 or PD0325902, even at high compound concentrations (Fig. 4B,C), indicating that MEK inhibition does not result in a complete cytostatic response in these cells. However, when BKM120 and PD0325901 were combined at equimolar concentrations, there was a 20% net decrease in viable cells at 48 h (and 70% at 72 h) compared to the 0 h time point, which indicates a clear cytotoxic response (Fig. 4D). Taken together, these results indicate that BKM120 and PD0325901 combination treatment induces a level of GBM cell death that is not observed with individual compound treatments.

To elucidate the mechanism of increased drug sensitivity with the combination treatment, we examined MBR614-2 cell apoptosis by using an ELISA that measures DNA fragmentation subsequent to programmed cell death (Fig. 4E). Treatment with either BKM120 or PD0325901 resulted in an increase in apoptosis after 24 h of treatment compared to that in DMSO-treated cells (twofold and fivefold respectively). After 48 h of treatment, the level of apoptosis was 1.5-fold higher with BKM120 and twofold higher for PD0325901 compared with that of DMSO-treated cells (Fig. 4E). However, when BKM120 and PD0325901 were combined, there was a significant increase in MBR614-2 cell death: up to 23-fold at 24 h and decreasing to fivefold at 48 h over the level of that of DMSO-treated controls. Thus, the cytotoxic response observed with this drug combination in cell proliferation assays is the result of an increase in apoptosis that is not seen with either agent alone.

### Combination treatment in the orthotopic model impedes GBM growth and improves overall survival

**Fig. 5.**
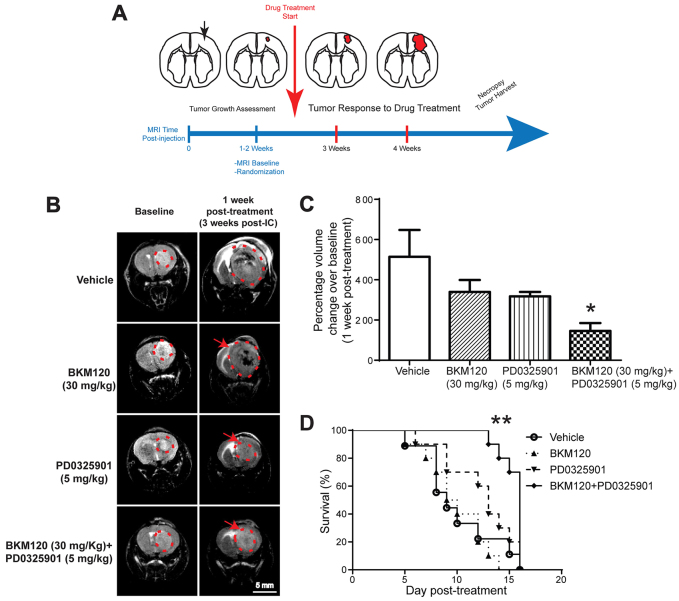
**Combined treatment of orthotopic GBMs with BKM120 and PD0325901 results in tumor growth delay and increased survival.** (A) Representative diagram of an efficacy study timeline conducted in the TRP orthotopic model. GBM-derived MBR525-1 primary cells were injected intracranially into strain-matched recipients. Weekly MRI scans were used to monitor tumor volume. Mice with established orthotopic tumors were randomized to different experimental groups for treatment. (B) Representative MRI scans at baseline and 1 week post-treatment. Red dashed lines indicate tumor location, and arrows show post-treatment tumors. (C) Percent change in tumor volume after 1 week of treatment. Data are represented as mean+s.d. (*n*=10, *P<0.01). Tumor growth inhibition was significant only in the combination treatment cohort. (D) Kaplan–Meier survival curve of mice treated as described in B and C (*n*=10, **P<0.001). Only the drug combination extended lifespan over that of vehicle-treated mice.

Characterization of our TRP orthotopic model revealed its relevance to human disease; therefore, we assessed whether it could be used to evaluate drug regimens that showed promise *in vitro*. TRP GBM tumor-derived MBR525 cells were injected intracranially into syngeneic wild-type mice, and magnetic resonance imaging (MRI) was performed weekly in order to monitor tumor development and the response to treatment (Fig. 5A). Tumors were detectable in the brain at 1 to 2 weeks post-intracranial cell injection, and mice were recruited and randomized into treatment cohorts by tumor volume. Orthotopic tumors exhibited rapid growth, so the number of primary cells injected was titrated to increase mouse survival to 6-weeks post-injection, thus allowing a longer time for therapeutic evaluation in efficacy studies.

Given the potency of BEZ235 and its inhibition of the PI3K pathway *in vitro* (supplementary material Fig. S3A), we expected that treatment would suppress tumor growth in the orthotopic model, as described previously in xenograft studies where BEZ235 efficacy was assessed (Liu et al., 2009). However, at the maximum dose (15 mg/kg of bodyweight) that was well-tolerated in our orthotopic GBM model, survival was not significantly increased compared with that of vehicle-treated mice (median survival of 9 and 11 days in vehicle- and BEZ235-treated groups, respectively) (supplementary material Fig. S3C).

Correspondingly, there was no change in tumor volume following BEZ235 treatment (supplementary material Fig. S3D). The PI3K pathway targets p-AKT, p-p70S6K and p-S6 were not consistently reduced in tumors (supplementary material Fig. S3E), indicating incomplete target suppression at this dose level (15 mg/kg). It is possible that the influence of the microenvironment in our syngeneic immunosufficient mice contributed to the limitations on dosing; recently, chronic dosing of BEZ235 and other PI3K inhibitors in wild-type mice at 10 mg/kg was shown to result in some toxicity, including animal death (Smith et al., 2013).

GBM cells were also sensitive to the PI3K-MEK inhibitor combination *in vitro*; therefore, we evaluated the efficacy of combination treatment in the tumor model. GSK1120212 was a potent MEK inhibitor in our GBM cells, but previous studies have shown that it does not accumulate in the brain (Gilmartin et al., 2011); therefore, we used PD0325901 for *in vivo* evaluation. At two weeks post-intracranial injection, mice were randomized by using tumor size into treatment groups (average tumor size per group=47 mm^3^) to receive vehicle, BKM120 (30 mg/kg), PD0325901 (5 mg/kg), or both drugs daily. Although these therapeutics have been given at higher doses when used as single agents (Brachmann et al., 2012; Henderson et al., 2010; Kinross et al., 2011; Maira et al., 2012; See et al., 2012), these dose levels were the maximum that were tolerated in combination in the GBM model (note that, prior to this study, we had evaluated additional combination regimens, including BKM120 at 60 mg/kg and PD0325901 at 10 mg/kg, that were not well-tolerated in brain-tumor-bearing mice). To measure the quantitative effect of treatment on tumor growth, baseline MRI-derived tumor volumes were compared with tumor scans after one week of treatment (Fig. 5B). Neither BKM120 nor PD0235901 alone significantly inhibited tumor growth relative to that of vehicle-treated mice; however, in the combination treatment cohort, tumor growth was inhibited by greater than 70% relative to that of vehicle-treated mice after one week of treatment (Fig. 5C).

Correspondingly, neither BKM120 nor PD0325901 significantly improved mouse survival (9.5 and 13 days median survival, respectively, compared with 9 days median survival for vehicle-treated mice). However, combination therapy with BKM120 and PD0325901 led to a survival benefit, with median survival extended to 16 days (Fig. 5D). Thus, enhanced pathway suppression that is induced by the combined targeting of PI3K and MEK pathways in TRP GBM cells was predictive of *in vivo* tumor growth inhibition.

### Combined inhibition of PI3K and MAPK enhances apoptosis in GBM tumors

**Fig. 6.**
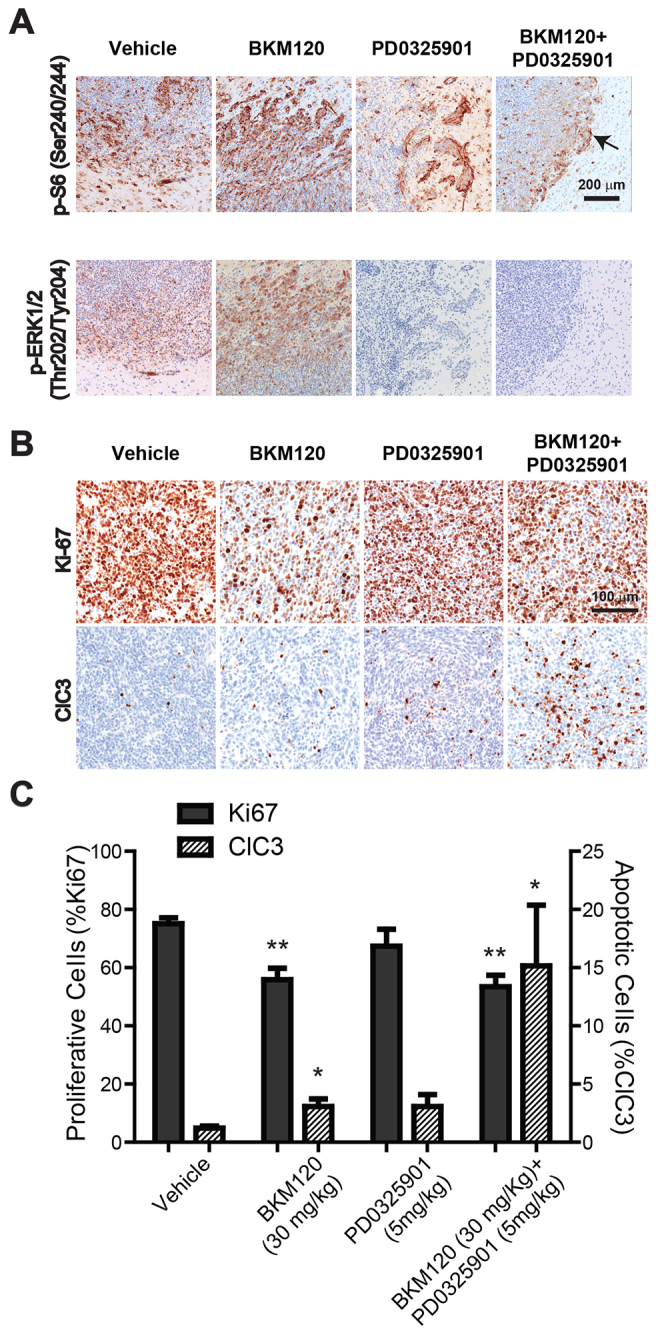
**Inhibition of both PI3K and MAPK pathways inhibits cell proliferation and increases apoptosis in GBM tumors.** Mice with orthotopic tumors were treated with vehicle (*n*=9), BKM120 at 30 mg/kg (*n*=10), PD0325901 at 5 mg/kg (*n*=10) or both BKM120 and PD0325901 at 30 mg/kg and 5 mg/kg (*n*=10), respectively, daily until euthanasia and tumor harvest (4 h post-last dose). (A) Immunohistochemistry for p-S6 (Ser240/244) and p-Erk1/2 (Thr202/Tyr204) in tumor tissue. The greatest reduction in p-S6 resulted from the combined treatment. p-S6 was inhibited by drug treatment mainly in the central regions of tumors, but expression was not suppressed at the invasive tumor periphery (arrow). (B) Immunohistochemistry staining for cell proliferation (Ki67) and cell death (cleaved caspase 3, ClC3). (C) Automated quantification of Ki67- and ClC3-positive cells in GBMs as described in Materials and Methods. Data are represented as mean+s.d. bars (*P<0.01, **P<0.001). The combined treatment resulted in a fivefold increase in apoptotic tumor cells over that of either single drug treatment, and a tenfold increase over that of vehicle-treated cells.

Pathway modulation as a result of treatment, and downstream effects on cell proliferation and apoptosis were examined in post-treatment tumor tissue by IHC. p-ERK (residues Thr202 and Tyr204) was reduced in response to treatment with PD0325901 (at 5 mg/kg) as expected, but p-S6 (residues Ser240 and Ser244) was reduced only in the combination BKM120-PD0325901-treated tumors (at 30 mg/kg and 5 mg/kg, respectively; Fig. 6A), indicating that, at this dose level, the PI3K pathway is not fully suppressed by BKM120 alone. In addition, histochemical analysis of the treated tumor tissue indicated that p-S6 was inhibited by drug treatment mainly in the central regions of tumors, but expression was not suppressed at the periphery of the invasive tumor (Fig. 6A). Cell proliferation (detected by IHC for Ki-67 followed by automated quantification) was reduced to an equal extent in BKM120-treated tumors and with the combination treatment, but not in PD0325901-treated tumors alone (Fig. 6B,C). An increase in apoptosis, detected by using cleaved caspase 3 (ClC3) immunostaining, was observed in BKM120-treated tumors compared with that of controls (twofold increase), but the dual treatment resulted in a marked greater effect, inducing a fivefold increase in apoptotic tumor cells over those exposed to the single drug treatment and a tenfold increase compared with that of vehicle-treated cells (Fig. 6B,C). The decrease in proliferation, together with an increase in apoptosis in combination-treated tumors, is consistent with tumor growth inhibition and the improved survival observed in BKM120-PD0235901-treated mice.

Because both drugs were given at sub-maximum tolerated dose (MTD) levels for the TRP orthotopic model in order to avoid toxicity of the combination treatment, we also examined the effects of the single agents in tumors at the MTD. PD0325901 given at 10 mg/kg did not induce a greater level of apoptosis compared to the 5 mg/kg dose, and the effect on cell proliferation was similar at both dose levels (supplementary material Fig. S4B). However, BKM120 treatment at 60 mg/kg resulted in both a greater decrease in cell proliferation and a larger increase in apoptosis than the 30 mg/kg dose used in combination studies (supplementary material Fig. S4B; Fig. 6B,C). Importantly, neither single agent given at the higher dose resulted in an increase in apoptosis equal to the levels we observed in tumors after combination treatment.

Although p-ERK (residues Thr202 and Tyr204) was reduced in response to treatment with PD0325901 (at 10 mg/kg), p-S6 (Ser240/244) was not significantly reduced in tumors that had been treated with the higher dose of BKM120 (at 60 mg/kg) (supplementary material Fig. S4C).

These findings confirm that the combined effect of PI3K and MEK inhibition allows for greater effects on cell proliferation and apoptosis in tumors in the context of a more tolerable dose regimen.

### MEK and PI3K inhibition together results in synergistic effects on downstream targets in brain tumors

**Fig. 7.**
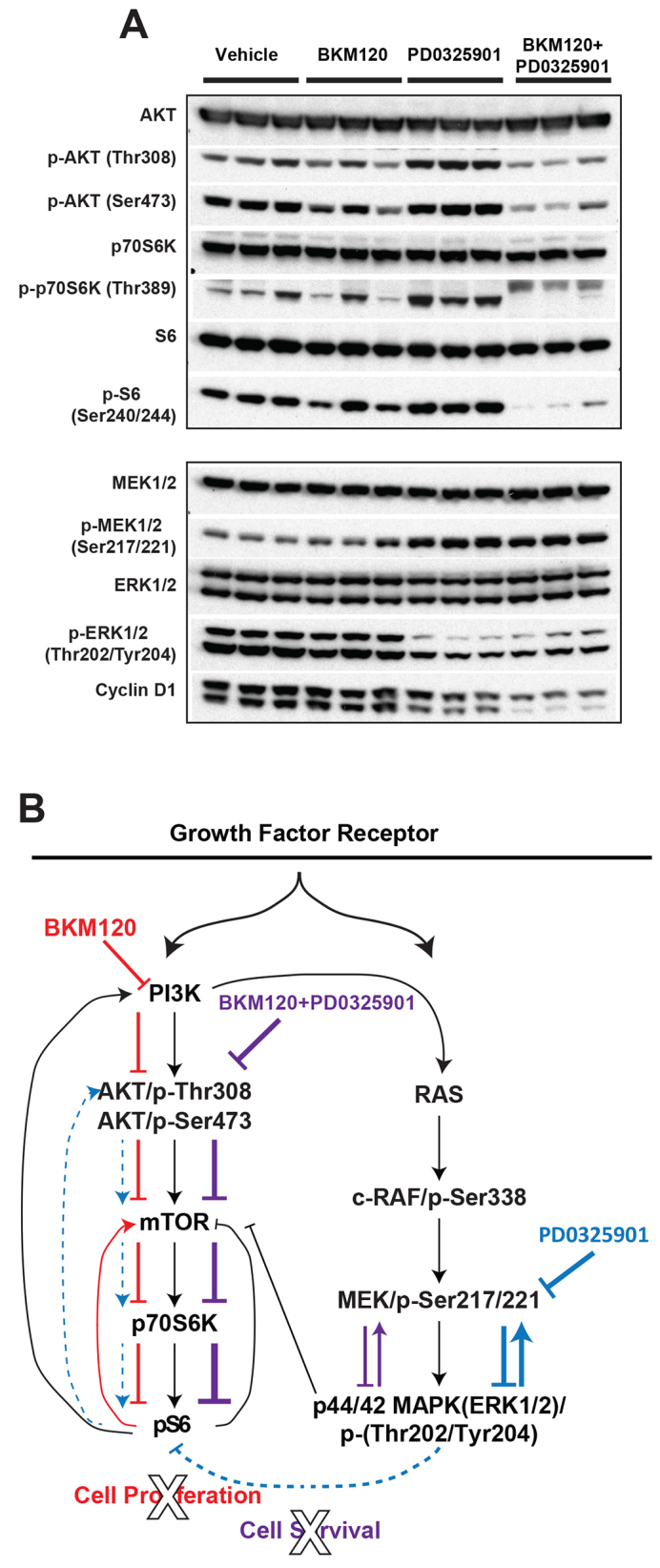
**Effect of therapy on tumor biomarkers.** (A) Tumor tissues from each treatment group were subjected to western blot analysis to examine markers in both the PI3K and MAPK pathways. The representative blot includes mice euthanized after at least 12 days of treatment with vehicle, at least 12 days of treatment with BKM120 (at 30 mg/kg), and 15 and 16 days of treatment with PD0325901 (at 5 mg/kg) alone or combined with BKM120 (at 30 mg/kg), respectively. BKM120-PD0325901 dual treatment suppressed p-ERK, and p-AKT, p-p70S6K and p-S6 were inhibited to a greater degree than with BKM120 alone, whereas total AKT, S6 and ERK were unchanged. (B) Diagram illustrating the PI3K and MAPK pathway responses in the GBM orthotopic model to BKM120 (red) and PD0325901(blue; dashed blue line indicates indirect effect on PI3K pathway), and the combined effect of both inhibitors (purple). Established PI3K and MAPK pathways are indicated (in black).

To correlate efficacy of the BKM120-PD0325901 combination to the activation status of target proteins, tumor tissue from the treatment study was examined for the phosphorylation status of proteins in both the PI3K and MAPK pathways. In BKM120-treated tumors, the levels of p-AKT at residues Tyr308 and Ser473 were decreased in response to BKM120; however, downstream targets pp70S6K and p-S6 were not consistently reduced (Fig. 7A). In PD0325901-treated tumors, the level of p-ERK was reduced, but with a corresponding increase in the level of p-MEK (Fig. 7A), consistent with the *in vitro* results after treatment with both PD0325901 and GSK1120212 (Fig. 3). Interestingly, cyclin D1 expression was reduced as well, suggesting a connection between ERK downregulation and cell cycle proteins downstream of PI3K, which has been previously noted in NF1-deficient GBM cells (See et al., 2012). BKM120-PD0325901 dual treatment suppressed p-ERK, and both p-AKT and p-p70S6K were inhibited to a greater degree than with BKM120 alone (Fig. 7A). Moreover, p-S6 and cyclin D1 were fully inhibited by the combination, indicating a synergistic effect on the PI3K pathway.

Taken together, these data indicate that even at sub-MTD doses, treatment with BKM120 and PD0325901 in combination results in a synergistic effect on the suppression of the PI3K pathway that potentiates a decrease in tumor cell proliferation and an increase in apoptosis. Direct inhibition of mTOR kinase can induce PI3K-AKT signaling by relieving mTOR-dependent feedback inhibition (Hu et al., 2005; Rodrik-Outmezguine et al., 2011). We propose that, in our orthotopic model, direct inhibition of PI3K also allows for feedback stimulation of downstream PI3K pathway components; however, the addition of a MEK inhibitor not only blocks feedback induction but enhances suppression of p-S6, either directly or, possibly, through ERK-mTOR pathway cross-talk (Fig. 7B) (Carracedo et al., 2008; Sunayama et al., 2010). Thus, this model is a useful tool for refining the most effective combinatorial drug regimens.

## DISCUSSION

Given the devastating nature of GBM in the clinic and the lack of effective therapies, a preclinical model is needed that recapitulates both the histopathological and molecular features of human GBM, and is conducive to the analysis of drug efficacy within a reasonable time frame (Hambardzumyan et al., 2011; Huse et al., 2013). GBMs are highly proliferative and infiltrative tumors with widespread microvascular proliferation and areas of focal necrosis (Wen and Kesari, 2008), and standard xenograft models lack the key histologic features that are characteristic of human GBM, as well as the immune system component (Becher and Holland, 2006). The orthotopic mouse model described here allows for the examination of the effects of targeted therapies on the pathways that are most prominently perturbed in GBM, as well as the therapeutic effects on tumor size and invasiveness, in the context of an intact immune system. Remarkably, the highly proliferative, invasive and vascular features of the TRP GEM model for GBM are recapitulated in the orthotopic tumors.

Based on the expression of signature genes, human GBMs have been recently classified into four functional subtypes: proneural, neural, classical and mesenchymal (TCGA, 2008; Verhaak et al., 2010). The TRP model described here can be placed into the classical or mesenchymal subtypes based on the engineered perturbations of RB, PTEN and KRAS, the latter resulting in an activated MAPK pathway. However, using tumor staining for Olig-2, Nestin and GFAP, we demonstrated that our preclinical model represents a range of GBM cell types, therefore modeling the heterogeneous cell population in human GBM. Additional molecular analysis of the TRP-orthotopic GBM model will be necessary in order to analyze specific gene expression signatures for comparison with the classification of human GBM.

Multiple mutations and redundant signaling pathways drive tumor growth in GBM; therefore, a model that represents a single driver mutation or pathway is less likely to represent drug sensitivity and resistance in human tumors. Although human GBMs frequently harbor high-level amplification of more than one RTK gene, our model is relevant for the evaluation of therapies for various subtypes, as downstream targets common to these receptors (Snuderl et al., 2011) are activated in GFAP-expressing cells. Other models with specific RTK activating events, such as the PDGF+PTEN−/−p53−/− mouse (Lei et al., 2011; Sonabend et al., 2013), more closely represent the events found in proneural GBM and are important tools for the evaluation of receptor-targeted therapeutics. The orthotopic GBM described in this report incorporates RB suppression, PI3K and MAPK pathway activation, thus representing the deregulation of core pathways common to most GBM tumors. Therefore, it is an optimized preclinical model to support the use of downstream pathway inhibitors for treatment, even in the case of the activation of EGFR, PDGFR, cMET or another RTK.

Activation of mutant RB or functional loss of RB and PTEN in the TRP orthotopic model mimicked the alterations observed in human GBM, and allowed us to examine the relative importance of inhibiting the PI3K and MAPK pathways *in vitro* and *in vivo* using drugs that are currently in clinical trials for the treatment of GBM (Bendell et al., 2012; LoRusso et al., 2010). BEZ235 has been shown to be effective in xenograft models for glioblastoma at higher doses (Liu et al., 2009); however, we were unable to dose at a level required to achieve either inhibition of PI3K and mTOR targets or a survival effect. Targeting both PI3K and mTOR has been shown to be essential for the elimination of feedback activation in glioma cells, and results in the inhibition of glioma cell proliferation (Fan and Weiss, 2006). Although combined targeting of multiple signaling pathways with a single drug is ideal, our study indicates that it might be necessary to separately dose inhibitors of each pathway so that both efficacy and tolerability can be maximized. Clearly, the involvement of other survival pathways in response to PI3K inhibition results in the need to suppress additional targets, as reported previously (Cheng et al., 2009). The evaluation of clinical results from dual targeting of the PI3K-AKT-mTOR and RAS-MEK-ERK pathways in individuals with cancer has shown the potential for efficacy but also greater toxicity (Shimizu et al., 2012). We found that with PI3K (BKM120) or MAPK (PD0325901) inhibitors alone, cell proliferation *in vitro* was reduced, but cytotoxicity was not observed. Cytotoxicity was observed in growth inhibition and apoptosis assays only when the drugs were combined. Correspondingly, a substantial increase in apoptosis in GBM tumors, along with decreased cell proliferation, was detected only in the BKM120-PD0325901 combination treatment group. The combination of pathway inhibitors resulted in an overall significant increase in survival over that of vehicle-treated mice which was not observed with either agent alone, indicating potential synergistic effects.

Previous studies have demonstrated the efficacy of MAPK and PI3K inhibitors in KRAS- and PI3K-driven mouse models of lung and ovarian cancer (Engelman et al., 2008). Others have reported *in vitro* sensitivity to PD0325901 and PI3K inhibitors in cells derived from mouse GBM models (Robinson et al., 2011). Our *in vivo* data further shed light in a translational context on the target effects that result in improved efficacy with dual treatment and uncover potential limitations on the dosing regimen that might affect strategy for the design of clinical therapeutic studies. BKM120 alone incompletely suppressed p-AKT and p-S6 in treated tumors while maintaining the p-AKT Ser473 to p-AKT Thr308 ratio, suggesting that mTORC2 negative feedback from p-S6 reduction stimulates the expression level of p-AKT Ser473 (Fig. 7B). This incomplete suppression of the PI3K pathway is consistent with the lack of apoptosis observed with single agent PI3K inhibitors and the necessity for combination treatments in order to achieve the cytotoxicity that has been reported in other studies (Cheng et al., 2009). PD0325901 alone significantly reduced the levels of p-ERK in tumors as expected, but with a corresponding increase in p-S6 and p-AKT, indicating cross-talk between the pathways. Importantly, inhibition of the PI3K pathway was substantially improved with the BKM120-PD0325901 combination, both *in vitro* and *in vivo*, as evidenced by a more complete suppression of p-S6. This cooperative effect might be due to abolition of the feedback activation of AKT signaling that is observed with either drug alone; when both drugs are present, PD0325901 depletes p-ERK, which results in indirect suppression of p-S6, possibly through the TSC1/2 or p90RSK complexes (Fig. 7B) (Carracedo et al., 2008). It is possible that using an additional p-S6 inhibitor would result in a further synergistic effect, as observed in NRAS-driven melanoma xenograft models (Posch et al., 2013); however, combination p-AKT-p-S6 inhibitors, such as BEZ235, have also been shown to limit synergistic growth inhibition when combined with MEK inhibition (Haagensen et al., 2012). Given the complexity of feedback regulation, the use of individual pathway inhibitors to evaluate combination dosing regimens *in vivo* would be more desirable.

In conclusion, we have developed an orthotopic mouse model of human GBM for preclinical studies that is beneficial to the evaluation of targeted therapies. Alterations in the PI3K and Kras pathways model oncogenic changes in human GBM, and we demonstrate that the combination of PI3K and MEK inhibitors have the potential to control GBM tumor growth and to extend survival. Given the aggressive nature of GBM that is reflected in this model, future studies could evaluate the standard of care chemotherapy and/or radiation in combination with targeted therapeutics. Additionally, the invasive nature of the tumors allows for the evaluation of potential suppressors of the tumor infiltration and migration that are characteristic of human disease.

## MATERIALS AND METHODS

### TRP mice

The ‘TRP’ mouse model expresses T121 (T) under the control of GFAP after Cre-mediated recombination (Miller et al., 2009; Schmid et al., 2012). T121 is a fragment of the SV40 antigen that inactivates the RB tumor suppressor, as well as compensatory proteins p107 and p130 (Song et al., 2013). To model RTK(s) activation (TCGA, 2008) in GBM tumors, the model expresses a constitutively active mutant *KRAS* allele (*KRAS*^G12D^; R) (Jackson et al., 2001; TCGA, 2008) that drives proliferation of astrocytoma. In addition, the mice are heterozygous for loss of PTEN (P) (Guha et al., 1997; Holland et al., 2000), which promotes cell survival and invasion and has been shown to accelerate progression to the GBM grade IV astrocytoma (Miller et al., 2009; Schmid et al., 2012; Song et al., 2013). Genetic events were induced in 12-week-old TRP mice through intraperitoneal injection of 4-OH-tamoxifen (4OHT, Sigma, St Louis, MO; dissolved 90:10 in sunflower oil:ethanol at 10 mg/ml) at 1 mg/mouse/day for five consecutive days (Song et al., 2013). Mouse breeding and procedures were conducted under an approved Animal Study Protocol according to Frederick National Laboratory Animal Care and Use Committee guidelines.

### Primary tumor cell culture

Mouse brain tumor primary cells (MBR) were isolated from Tamoxifen-induced TRP GEM mice. GBM was confirmed on the remaining tissue by histopathology. Approximately 3–4 months post-induction, tumor tissue was harvested and dissociated in 0.05% Trypsin-EDTA (Life Technologies). Cells were grown at 37°C under 5% CO_2_ in Dulbecco’s modified Eagle’s medium (DMEM) supplemented with 10% fetal bovine serum, 0.1 mg/ml streptomycin, 100 units/ml penicillin. Primary cells were passaged at least twice *in vitro* prior to *in vivo* use. MBR614-2 and MBR525-1 cell data were included in this report.

### Cell viability

MBR cells were plated at 5000 cells per well in 96-well plates. Cells were allowed to adhere for 16 h before the medium was replaced with drug-containing medium. Unless otherwise indicated, single drug treatment was conducted at a range of concentrations between 0.1 and 50,000 nM in 0.5% DMSO; controls in each plate included DMSO only, 200 nM and 800 nM Doxorubicin and 0.1% Triton. Drug-treated and control wells were run in triplicate. At 72 h after drug exposure, cell viability was measured using XTT Assay (Roche) as per the manufacturer’s instruction. DMSO-treated wells were considered as 100% viability for each treatment plate.

### Orthotopic mouse model

Nine-week-old B6D2F1/J mice purchased from The Jackson Laboratory (Bar Harbor, ME) were used to develop an orthotopic mouse GBM model using primary cells dissociated originally from TRP GEM GBM tumors as described above. Dissociated MBR cells were washed in serum-free medium, counted and resuspended in 5% methylcellulose (Sigma). MBR cells at 5×10^4^ in 2 μl volume were injected intracranially with a stereotactic apparatus using the following coordinates: 3 mm posterior, 2 mm lateral right to the bregma and 2 mm deep from the dura. Mice were monitored daily for symptoms and screened for tumor development with *in vivo* imaging (see MRI below). Brain tumors were allowed to grow at least 2 to 3 weeks before recruiting mice into efficacy study groups. Mice were observed daily for signs of brain tumor growth, such as seizures, ataxia or weight loss, and were euthanized when tumor burden became symptomatic. At the time mice bearing brain tumors were enrolled in efficacy studies, they exhibited no clinical signs.

### Drug treatments

NVP-BEZ235 (BEZ235), NVP-BKM120 (Buparlisib, BKM120), GSK1120212 (Trametinib, JTP-74057), PD0325901, and AZD6244 were purchased from Chemie Tek (Indianapolis, IN). Temozolomide, doxorubicin and paclitaxel were purchased from Sigma. For *in vivo* studies, BEZ235 and BKM120 were resuspended in NMP (N-methyl-2-pyrrolidone):PEG300 1:9 v:v, and GSK1120212 and PD0325901 were resuspended in 0.5% hydroxypropyl-methylcellulose (Sigma):0.2% Tween 80 (Sigma) (Gilmartin et al., 2011; Kinross et al., 2011). BKM120, GSK1120212 and PD0325901 were given once daily by oral gavage at 10 ml/kg. Mice were treated with BEZ235 on a 5 days on, 2 days off schedule (Liu et al., 2009). Vehicle- and drug-treated mice were closely monitored on a daily basis for clinical signs as described above.

### Apoptosis assay

Apoptosis was detected in vehicle- or drug-treated MBR cells with a cell death ELISA (Roche Diagnostics, IN). Briefly, cells (200,000 per time point) were treated with vehicle or the indicated drug for 24 or 48 h, and cell lysates were used for the detection of oligonucleosomes using an antibody against histone.

### *In vivo* imaging and analysis

Four mice were simultaneously imaged in the supine position on a MRI 3.0T clinical scanner (Philips Healthcare Intera Achieva, Andover, MA) with a custom-built four mouse SENSE array surface coil (Choyke et al., 2003). Multislice T2-weighted turbo spin echo (T2w-TSE) sequence: (TR/TE (4437/100 ms), in plane resolution (0.12×0.15 mm), slice thickness (0.5 mm), SENSE acceleration factor (4) images were acquired in the axial plane to cover the entire mouse brain. The volumes of the brain tumors were measured by using ITK_SNAP (Version 2.2.0, May 4, 2011) analysis of MRI images. After uploading an MRI image into the ITK_SNAP program, the image contrast was adjusted and the image intensity region filter was adjusted. Following the active contour initialization and image segmentation, the tumor volume was measured in mm^3^.

### Histopathology

Mouse brains were dissected and fixed in 10% neutral buffered formalin for 24 h and embedded in paraffin. Paraffin blocks were cut in serial sections, deparaffinized, rehydrated and prepared for hematoxylin and eosin (H&E) staining. H&E-stained, sagittal sections of the entire brain from all available mice were evaluated by an ACVP-board-certified veterinary pathologist (P.L.M.), and the tumors were graded based upon the current World Health Organization (WHO) classification for human astrocytomas (Raza et al., 2004; Raza et al., 2002).

### Immunohistochemistry and quantitative immunohistochemistry

Paraffin sections (5 μm) were prepared and subjected to either EDTA- or citrate-based antigen retrieval before staining with primary antibodies against the following proteins: T121 (1:100, Calbiochem), GFAP (1:1000, DAKO), Olig-2 (1:500, Millipore), Nestin (1:1000, Millipore), Sox-2 (1:300, Cell Signaling), Ki67 (1:500, Bethyl Labs), cleaved caspase 3 (ClC3, 1:100, Cell Signaling), p-S6 (Ser240/244) (1:2000, Cell Signaling) and p-ERK1/2 (Thr202/Tyr204) (1:100, Cell Signaling). Primary antibody incubation was performed for 1 h at room temperature on a Leica BondMax autostainer (Leica, IL) utilizing the Polymer Refine kit (Leica, IL) for the following markers: GFAP, Sox-2, Ki67, CLC 3, p-S6 (Ser240/244) and p-ERK1/2 (Thr202/Tyr204). The remaining markers were stained manually with antigen retrieval in a pressure cooker followed by primary incubation overnight at 4°C. Secondary antibody incubation was performed at room temperature for 30 min utilizing a horseradish-peroxidase (HRP)-labeled rabbit IgG on rodent polymer (Biocare Medical, Concord, CA), and staining was visualized with DAB (Sigma Fast DAB tablets, Sigma) for 10 min at room temperature. All slides were counterstained with hematoxylin, dehydrated and permanently mounted. For quantitative immunohistochemistry, slides were scanned and analyzed using the Ariol™ scanning and analysis system. For automated quantification of Ki67 and cleaved caspase 3, the kisight analysis module was used to quantify positive nuclei (DAB-brown) and negative nuclei (counter-stained blue with hematoxylin). For automated quantification, the entire orthotopic tumor was selected for analysis to avoid any sampling bias. This resulted in ~10,000 to 200,000 cells quantified per tumor.

### Western blot

MBR cells or brain tumor samples were resuspended or homogenized in tissue and cell lysis buffer [50 mM Tris, 150 mM NaCl, 1 mM EDTA, 1% NP40, 10% glycerol, 1 mM Na_3_VO_4_, 1 mM DTT, 1 mM PMSF, 1× protease inhibitor (Sigma), 1× phosphatase inhibitors (Sigma)], rotated at 4°C for 30 min and centrifuged at 13,000 r.p.m. for 10 min at 4°C. Protein concentration was determined by using BCA assay (Thermo Scientific-Pierce). Western blot analyses were conducted after separation by SDS-PAGE electrophoresis and transfer to nitrocellulose membranes. Immunoblotting was performed according to the antibody manufacturers’ recommendations. Primary antibodies were obtained from Cell Signaling: p-AKT (Thr308 and Ser473), AKT, p-p70 (Thr389), p70, p-S6 (Ser240/244), S6, p-C-Raf (Ser338) and (Ser259), C-Raf, p-MEK1/2 (Ser217/221), MEK1/2, p-Erk1/2 (Thr202/Tyr204), and Erk1/2. β-actin (Sigma) was used for gel loading control.

## Supplementary Material

Supplementary Material
