# Supplementary material for: A preclinical orthotopic model for glioblastoma recapitulates key features of human tumors and demonstrates sensitivity to a combination of MEK and PI3K pathway inhibitors
Source: Dis Model Mech. 2014 Nov 27;8(1):45–56. doi: 10.1242/dmm.018168 (PMC4283649; doi:10.1242/dmm.018168)
Supplement: Supplementary Material [file supp_8_1_45__index.html]

A preclinical orthotopic model for glioblastoma recapitulates key features of human tumors and demonstrates sensitivity to a combination of MEK and PI3K pathway inhibitors — Supplementary Material 

# A preclinical orthotopic model for glioblastoma recapitulates key features of human tumors and demonstrates sensitivity to a combination of MEK and PI3K pathway inhibitors

## DMM018168 Supplementary Material

**Files in this Data Supplement:**

- **Supplementary Material**
