# Supplementary material for: A preclinical orthotopic model for glioblastoma recapitulates key features of human tumors and demonstrates sensitivity to a combination of MEK and PI3K pathway inhibitors
Source: Dis Model Mech. 2014 Nov 27;8(1):45–56. doi: 10.1242/dmm.018168 (PMC4283649; doi:10.1242/dmm.018168)
Supplement: Supplementary Material [file supp_8.1.45_DMM018168.pdf]

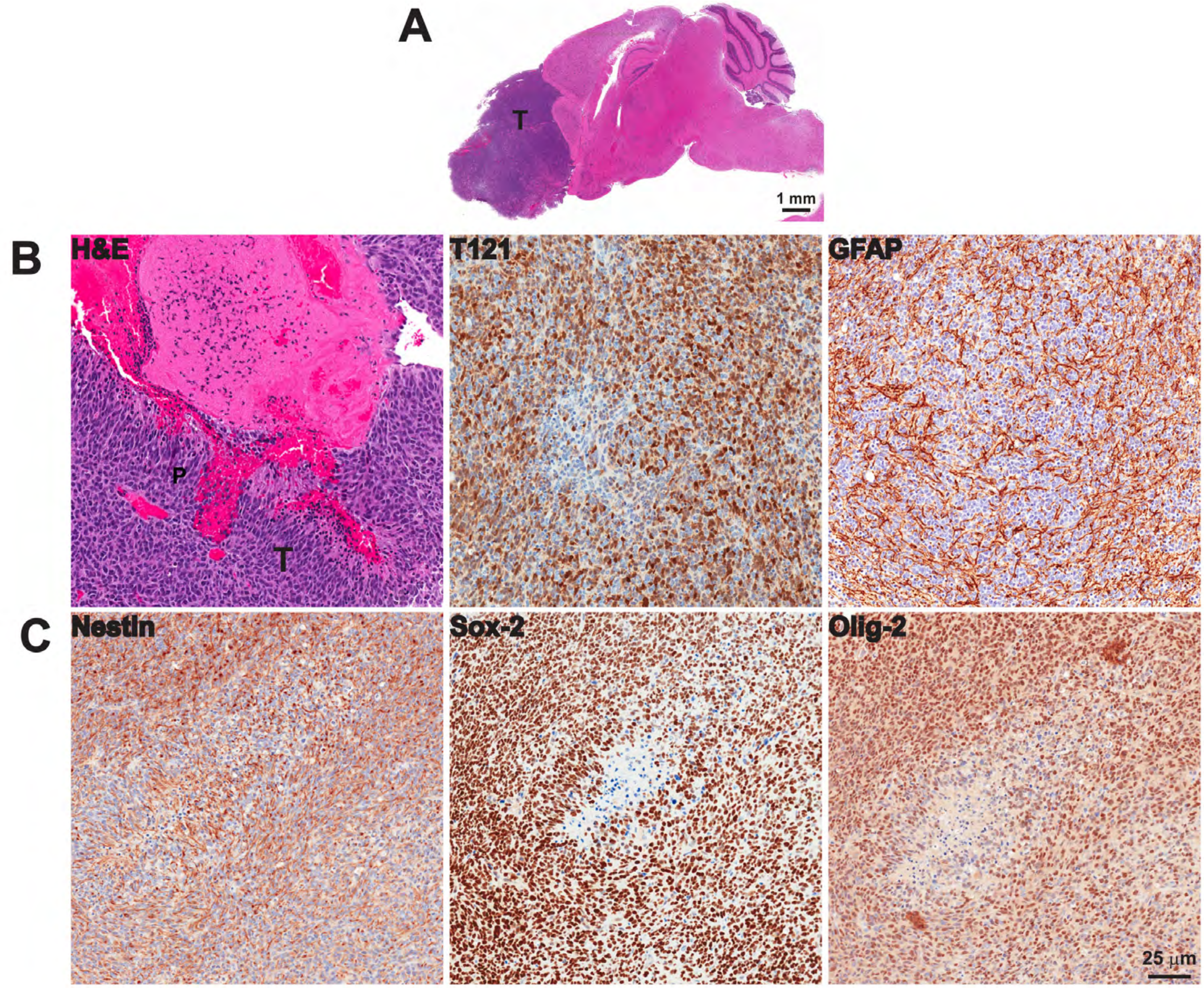

Fig. S1. Characterization of GBMs in TRP donor GEMs. TRP GBMs contain regions of necrosis and hemorrhage with pseudopalisading tumor cells (P). As in orthotopic GBMs, most cells express T121. GFAP expression is very heterogeneous with cells expressing high levels admixed with cells with no detectable expression. (T)=tumor region. Most neoplastic cells in TRP GBMs express Nestin, Sox-2, and Olig-2.

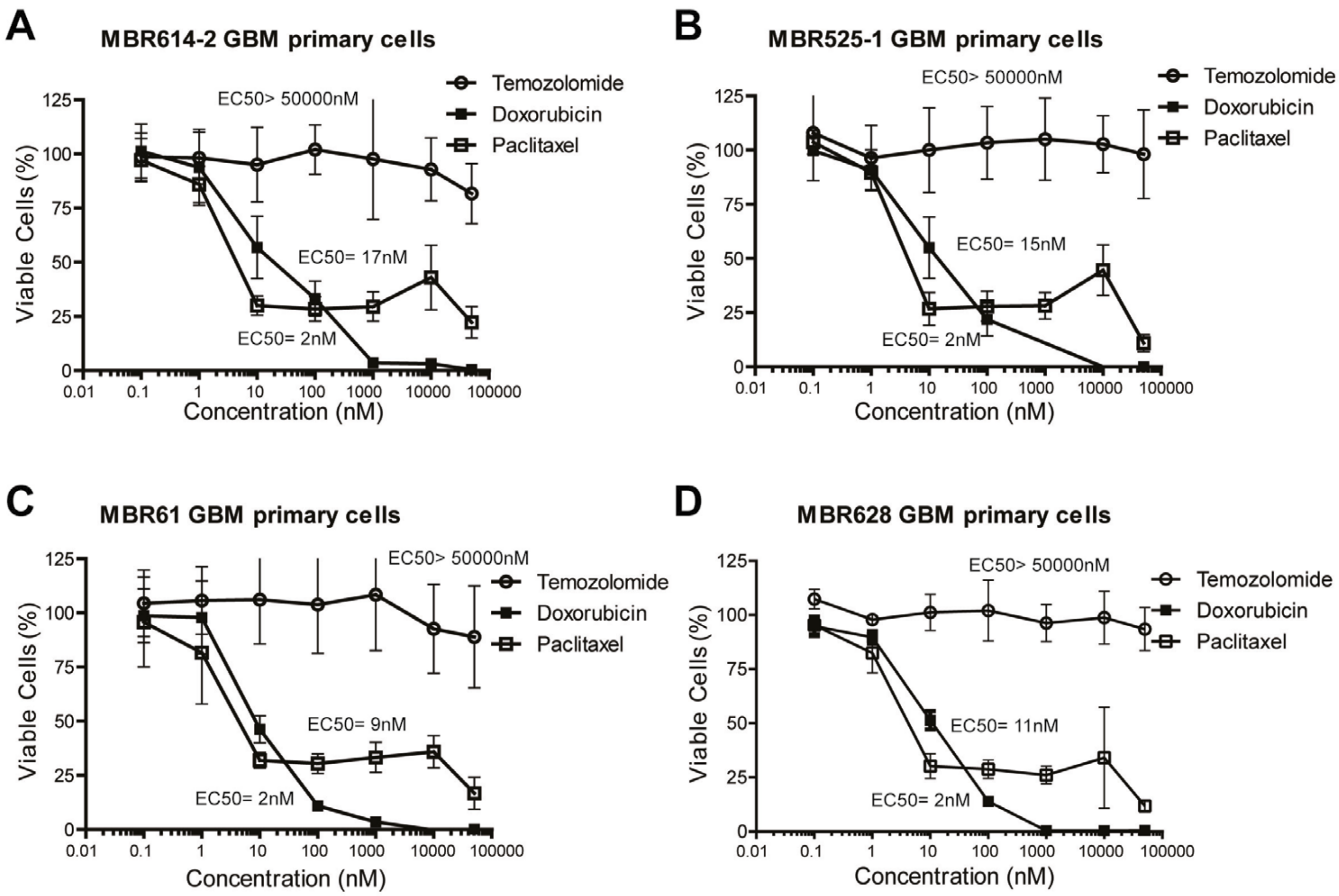

Fig. S2. Mouse GBM derived cells are insensitive to most standard of care chemotherapeutic agents. Primary GBM cells were treated with Temozolomide, doxorubicin, and paclitaxel for 72h and subjected to XTT assays as described in Materials and Methods. Curves show the percentage viability of chemotherapy-treated cells (A), MBR614-2; (B), MBR525-1; (C), MBR61; (D), MBR628) compared with the vehicle treated control cells. Each agent testing was repeated at least in 3 times in three independent experiments. Each dose treatment was done in triplicate, and bars represent s.d.

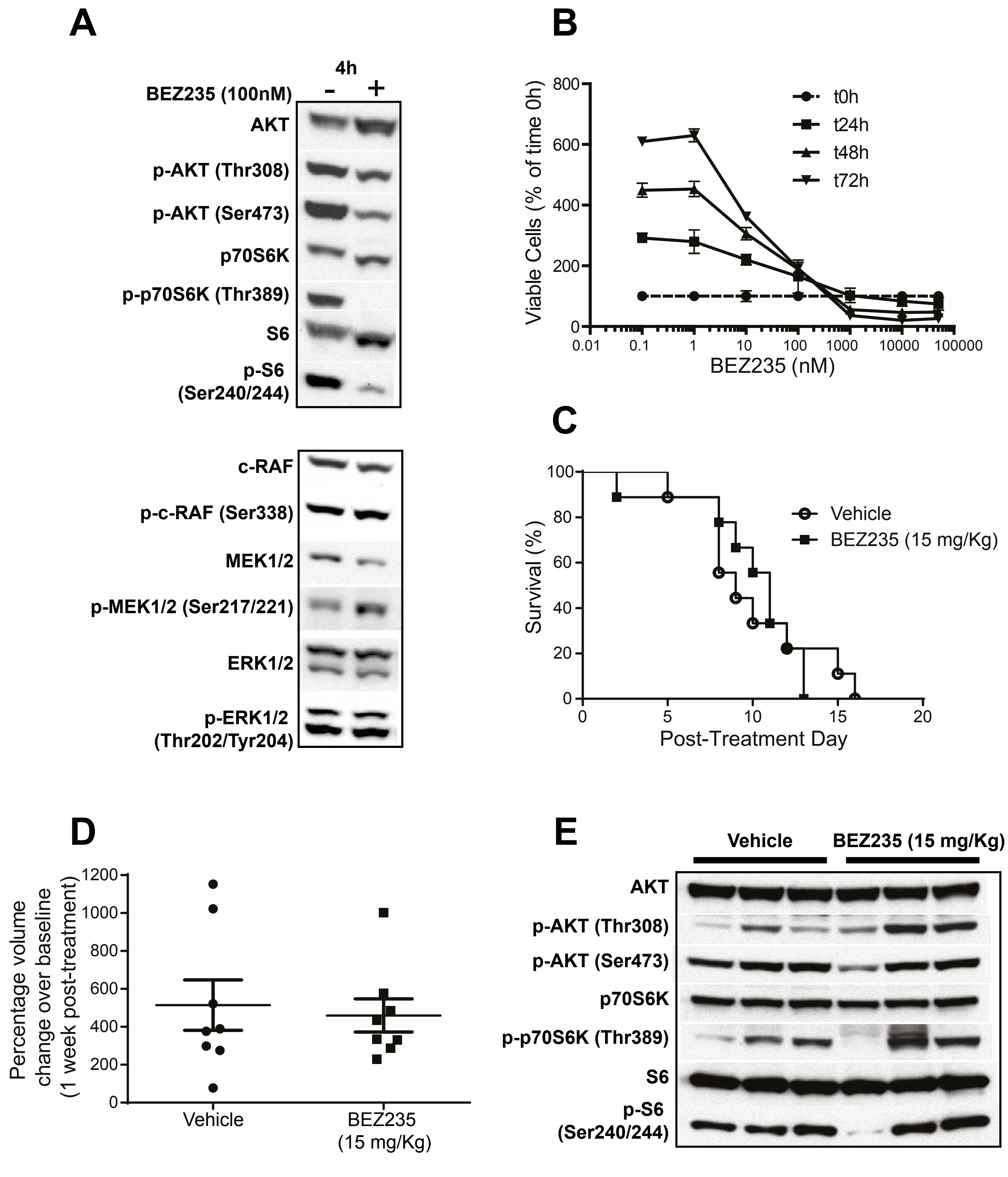

Fig. S3. Effect of BEZ235, a dual PI3K/mTOR inhibitor, on GBM cells and orthotopic tumors. (A) Immunoblot analysis of MBR614-2 cells treated with BEZ235. Cells were treated with DMSO control (-), or BEZ235 (+) at 1μM for 4h. BEZ235 treatment results in reduction of p-p70 S6K and p-S6, but does not fully suppress p-Akt. (B), Cells were exposed to the indicated drug concentrations for 24h, 48h, or 72h and cell growth/viability (by XTT assays) was determined by normalization to the viability at time 0h. Net cell death occurs by 48 hour. (C,D) Efficacy of BEZ235 in mice with orthotopic brain tumors. Mice were treated with vehicle (*n*=9), or BEZ235 (*n*=9) at 15 mg/Kg daily on a 5 days on/2 days off schedule until euthanisia due to tumor burden or clinical signs. (C), survival curve indicates no significant difference in survival between vehicle- and BEZ235-treated mice. (D),Tumor growth was not delayed in BEZ235-treated mice compared to vehicle-treated after 1 week of treatment. (E), Western blot of brain tumors from 3 vehicle- and 3 BEZ235-treated mice (15 mg/kg).

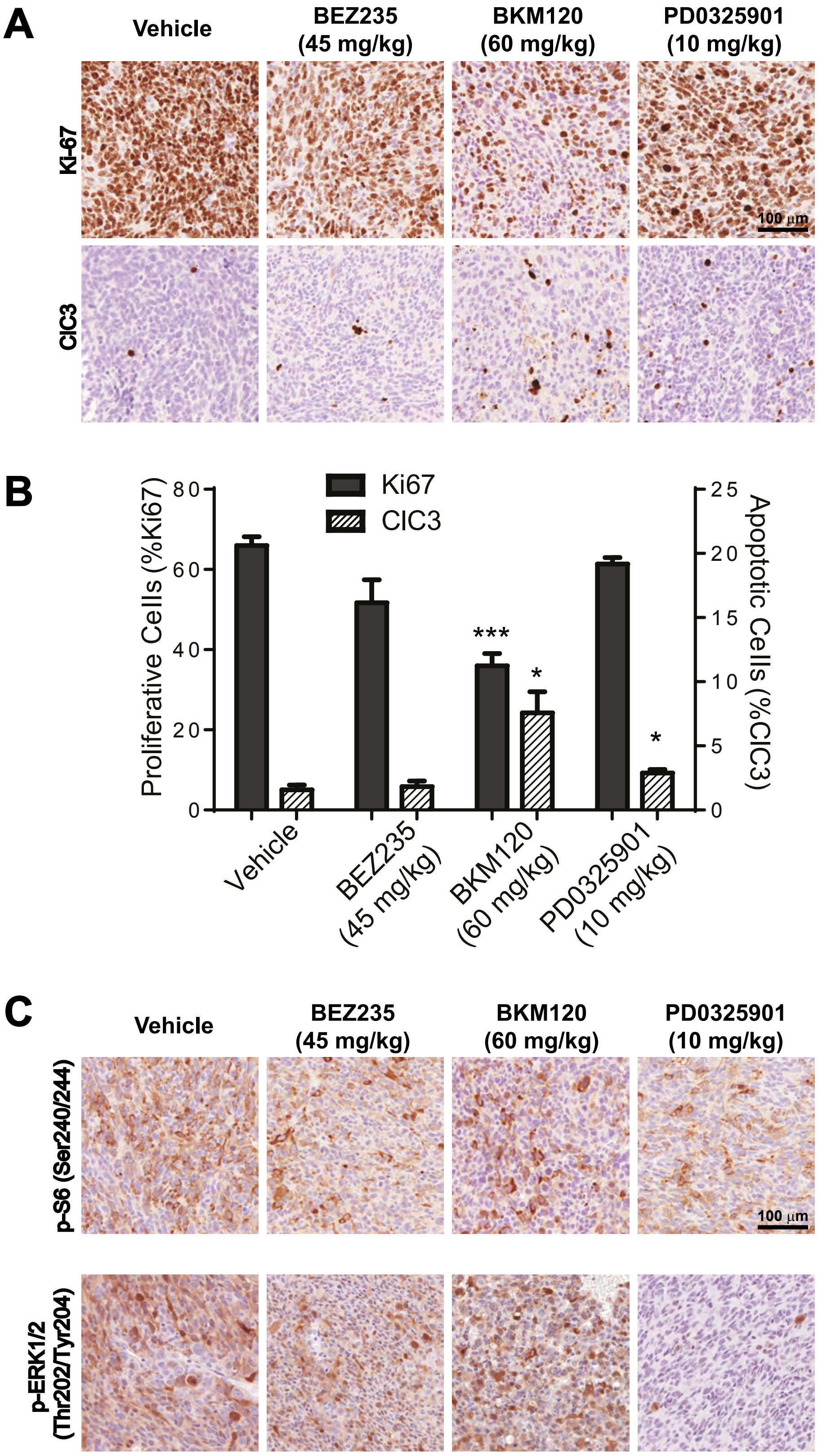

Fig. S4. Comparison of dual PI3K/mTOR inhibitor to a pan-PI3K inhibitor and a MEK inhibitor administered at the MTD level on GBM cell proliferation and apoptosis. Mice with established orthotopic tumors were treated with vehicle (*n*=11, daily for an average of 8 days), BEZ235 at 45 mg/kg (*n*=10, daily for an average of 4 days), BKM120 (*n*=10, daily for an average of 13 days) at 60 mg/kg, and PD0325901 at 10 mg/Kg (*n*=10, daily for an average of 12 days). (A), Following therapy tumors were analyzed for cell proliferation using Ki67 IHC and cell death using cleaved caspase 3 (ClC3) IHC. (B), shows automated quantification of Ki67 and ClC3 positive cells in GBMs harvested from the different treatment groups as described in Materials and Methods. Data are represented as mean with s.d. bars (*, $P<0.01$, and ***, $P<0.0001$). (C), IHC staining for p-S6 (S240/244) and p-Erk1/2 (T202/Y204) in tumor tissue from the high dose single treatment study.
